# Annual acknowledgement of manuscript reviewers

**DOI:** 10.1186/2045-4015-2-9

**Published:** 2013-03-27

**Authors:** Bruce Rosen, Avi Israeli

**Affiliations:** 1 Co-editors, Israel Journal of Health Policy Research (IJHPR), London, UK

## Abstract

**Contributing reviewers:**

The Editors of *Israel Journal of Health Policy Research* would like to thank all reviewers, both external and Editorial Board Members, who have contributed to the journal since its inception, and whose valuable support continues to be essential to the success of the journal.

## 

Julia Adler-Milstein

United States of America

Arnon Afek

Israel

Akke Albada

Netherlands

Yael Applbaum

Israel

Nachman Ash

Israel

Yael Ashkenazi

Israel

Toni Ashton

New Zealand

Uri Aviram

Israel

Alexander Aviram

Israel

Orna Baron-Epel

Israel

Arie Ben Yehuda

Israel

Jochanan Benbassat

Israel

Matthew Bending

United Kingdom

Netta Bentur

Israel

Gabi Bin Nun

Israel

Stephen Birch

United Kingdom

Linda Birnbaum

United States of America

Yair Birnbaum

Israel

Haim Bitterman

Israel

Gary Bond

United States of America

Heather Boon

Canada

Jeffrey Borkan

United States of America

Shuli Brammli-Greenberg

Israel

Mayer Brezis

Israel

Ronit Calderon-Margalit

Israel

Silvana Castaldi

Italy

David Chinitz

Israel

A Mark Clarfield

Israel

Robert Cohen

Israel

Marc Cohen

United States of America

Gregory Connolly

United States of America

Richard Cooper

United States of America

Janet Corrigan

United States of America

Yehuda Danon

Israel

Gordon DeFriese

United States of America

Milka Donchin

Israel

Robert Drake

United States of America

Asher Elhayany

Israel

Linda Emanuel

United States of America

Keren Eyal

Israel

Henry Falk

United States of America

Roger Feldman

United States of America

Penny Feldman

United States of America

Eric Finkelstein

Singapore

Gary Freed

United States of America

Yosef Frost

Israel

Guido Giarelli

Italy

Stanton Glantz

United States of America

Shimon Glick

Israel

Marsha Gold

United States of America

Melissa Goldstein

United States of America

Manfred Green

Israel

Dan Greenberg

Israel

Michael Grodin

United States of America

Itamar Grotto

Israel

Jonathan Halevy

Israel

Pinchas Halpern

Israel

Martin Hatlie

United States of America

Maria M. Hofmarcher-Holzhacker

Austria

Tuvia Horev

Israel

Orit Jacobsen

Israel

Jonathan Javitt

United States of America

Deborah Juarez

United States of America

Nir Kaidar

Israel

Ofra Kalter-Leibovici

Israel

Lital Keinan-Boker

Israel

Arthur Kellermann

United States of America

Ilona Kickbusch

Switzerland

Kristin Kiesel

United States of America

Barry Kinzbrunner

United States of America

Gerald Konrad

Canada

Ankit Kumar

France

Amnon Lahad

Israel

Bruce Landon

United States of America

Fleur-Ange Lefebvre

Canada

Moshe Leshno

Israel

Osnat Levtzion-Korach

Israel

Harold Luft

United States of America

Nicole Lurie

United States of America

William Maas

United States of America

David Mankuta

Israel

Idit Matot

Israel

Martin McKee

United Kingdom

Patriek Mistiaen

Netherlands

Shlomo Mizrahi

Israel

Lahad Muli

Israel

Shlomo Mor-Yosef

Israel

Denise Naon

Israel

Nurit Nirel

Israel

Gur Ofer

Israel

Ari Paltiel

Israel

Mical Paul

Israel

Christopher Pearce

Australia

Mark Pearson

France

Rosalind Plowman

United Kingdom

Phillippa Poole

New Zealand

Avi Porath

Israel

Vardit Ravitsky

Canada

Shmuel Reis

Israel

Shimon Reisner

Israel

Stephen Resch

United States of America

Lee Resnick

United States of America

David Rier

Israel

Laura Rosen

Israel

Joseph Rosenblum

Israel

Lisa Rubin

Israel

Richard Saltman

United States of America

Ami Schattner

Israel

Hillel Schmid

Israel

Stephen Schoenbaum

United States of America

Jonas Schreyögg

Germany

Robert Schwartz

Canada

Walter Sermeus

Belgium

Judith Shamian

Canada

Amir Shanon

Israel

Shmuel Shapira

Israel

Oren Shavit

Israel

Tamy Shohat

Israel

Pesach Shvartzman

Israel

Gil Siegal

Israel

Elisheva Simchen

Israel

Pierre Singer

Israel

Samantha Smith

Ireland

Alden Solovy

Israel

Katherine Swartz

United States of America

Yossi Tamir

Israel

Elizabeth Temkin

Israel

Lawrence Tsen

United States of America

Theodore Tulchinsky

Israel

Wynand van de Ven

Netherlands

Daniel Vardy

Israel

Kim Walsh-Childers

United States of America

Odette Wegwarth

Germany

Michael Weingarten

Israel

Ram Weiss

Israel

Yael Yishai

Israel

Itzhak Zaidise

Israel

